# First-Principles Investigation into the Interaction of H_2_O with α-CsPbI_3_ and the Intrinsic Defects within It

**DOI:** 10.3390/ma17051091

**Published:** 2024-02-27

**Authors:** Na Wang, Yaqiong Wu

**Affiliations:** 1 Department of Physical Chemistry, University of Science and Technology Beijing, Beijing 100083, China; 2 School of Metallurgical and Ecological Engineering, University of Science and Technology, Beijing 100083, China

**Keywords:** CsPbI_3_, H_2_O, vacancy, binding energy, stability

## Abstract

CsPbI_3_ possesses three photoactive black phases (α, β, and γ) with perovskite structures and a non-photoactive yellow phase (δ) without a perovskite structure. Among these, α-CsPbI_3_ exhibits the best performance. However, it only exists at high temperatures and it tends to transform into the δ phase at room temperature, especially in humid environments. Therefore, the phase stability of CsPbI_3_, especially in humid environments, is the main obstacle to its further development. In this study, we studied the interaction of H_2_O with α-CsPbI_3_ and the intrinsic defects within it. It was found that the adsorption energy in the bulk is higher than that on the surface (−1.26 eV in the bulk in comparison with −0.60 eV on the surface); thus, H_2_O is expected to have a tendency to diffuse into the bulk once it adsorbs on the surface. Moreover, the intrinsic vacancy of V_Pb_^0^ in the bulk phase can greatly promote H_2_O insertion due to the rearrangement of two I atoms in the two PbI_6_ octahedrons nearest to V_Pb_^0^ and the resultant breaking of the Pb–I bond, which could promote the phase transition of α-CsPbI_3_ in a humid environment. Moreover, H_2_O adsorption onto V_I_^+1^ contributes to a further distortion in the vicinity of V_I_^+1^, which is expected to enhance the effect of V_I_^+1^ on the phase transition of α-CsPbI_3_. Clarifying the interaction of H_2_O with α-CsPbI_3_ and the intrinsic defects within it may provide guidance for further improvements in the stability of α-CsPbI_3_, especially in humid environments.

## 1. Introduction

Over the last decade, the efficiency of organic–inorganic hybrid halide perovskite (HHP) solar cells has improved, and now ranges from 3.8% to 25.2% [1,2,3,4,5,6]. However, HHP solar cells are generally less stable due to the volatility and hygroscopicity of organic cations in the perovskite light-collecting layer [7,8,9]. The low stability of typical HHPs, such as MAPbI_3_ (MA^+^: methylammonium) and FAPbI_3_ (FA^+^: formamidine), has been noted since the early stages of perovskite solar cell (PSC) research [7,8,9]. In order to address the “vulnerability” of HHP in ambient air, organic components, including MA^+^ and FA^+^, have been partially or even completely replaced by Cs^+^ or Rb^+^ [10,11,12,13]. Completely inorganic halide perovskites (IHPs) show greater prospects for photoelectric applications because of their suitable optical properties and higher stability under external stimuli [14]. Among these, CsPbI_3_ is the most typical, with a lower production cost [13,15,16,17,18]. Furthermore, cubic α-CsPbI_3_ has a direct band gap [19], a wide absorption spectrum in the solar region, high quantum efficiency, and a long radiation life, meaning that it is expected to be an excellent candidate for use in perovskite solar cells [20]. However, three photoactive “black” perovskite phases (α, β, and γ) of CsPbI_3_ can be easily transformed into a more thermodynamically stable “yellow” non-perovskite phase (δ phase) under ambient conditions [21,22], and this polymorphic transformation becomes even more severe when water is present [16,23].

Various theoretical studies have revealed the possible degradation mechanism of CsPbI_3_ in a humid environment. The transformation of CsPbI_3_ from the α to δ phases has always been attributed to the lower formation enthalpy of the δ phase [16]. Moreover, the instability of the α phase of CsPbI_3_ has been well documented to be attributed to the phonon instability of α-CsPbI_3_ [24,25]. Kye et al. have further pointed out that the cation vacancy (V_Cs_ and V_Pb_) can weaken the interaction between Cs and PbI_6_ and may lower the nucleation barrier, thus promoting phase transformation [26]. On the other hand, Lin et al. have pointed out that I vacancies (V_I_) could reduce the surface tension between the α and δ phases and lower the nucleation barrier [23]. It has also been preliminarily determined that H_2_O induces a catalytic effect [16]. Jiang et al. [27] studied the effects of several air molecules and found that H_2_O may diffuse into and produce a polycrystalline structure and grain boundary, and eventually lead to the phase transformation of CsPbI_3_. Li et al. [28] have explored the counterpart system, CsSnI_3_, and found that the strong coupling between O and Cs and the hydrogen bond between H and I may lead to the deformation of the (001) surface and, thus, phase instability. Moreover, Lin et al. have also further elaborated that H_2_O adsorbed on the surface of α-CsPbI_3_ film may introduce V_I_, thus effectively catalyzing the transformation from the α to δ phases [23].

However, the exquisite interaction of H_2_O with CsPbI_3_ and the intrinsic defects within it have not yet been studied. Based on first-principles calculations, we first compared the interaction of H_2_O on the (001) surface and in the bulk of α-CsPbI_3_. It was found that H_2_O binds more strongly in bulk α-CsPbI_3_ than on the surface; thus, H_2_O tends to diffuse into the bulk. Moreover, we further analyzed the interaction between H_2_O and the neutral and charged intrinsic vacancies in α-CsPbI_3_. It was found that a neutral Pb vacancy V_Pb_^0^ may significantly accelerate the insertion of H_2_O. The strong binding between H_2_O and V_Pb_^0^ induces Pb–I bond breakage and new I–I bond formation, which are expected to promote the phase transition. Moreover, H_2_O adsorption onto V_I_^+1^ contributes to a further distortion in the vicinity of V_I_^+1^, which is expected to enhance the phase transition effect of V_I_^+1^. This work may provide guidance for the improved stability of CsPbI_3_.

## 2. Calculation

All the calculations were performed using the code of the Vienna ab initio simulation package (VASP) [29] within the density functional theory (DFT) framework [30]. The Perdew–Burke–Emzerh (PBE) [29] exchange–correlation function within the generalized gradient approximation (GGA) [31] method was used, and the plane wave cutoff energy was 450 eV. The optimized lattice constants of the α-CsPbI_3_ structure (pm3m) were a = b = c = 6.414 Å, which are consistent with the previous calculations (6.40 Å) and experimental measurements (6.18 Å) [32,33]. All of the atoms were fully relaxed, until the total energy per atom was less than 1 × 10^−6^ eV and the Hellmann–Feynman force per atom was less than 0.01 eV/Å. For adsorption on the α-CsPbI_3_ (001) surface systems, we expanded the unit cell into a 2 × 2 supercell in the ab plane and selected a K-mesh size of 3 × 3 × 1; for the insertion of H_2_O into the bulk phase of α-CsPbI_3_, we constructed a 3 × 3 × 3 supercell and used a 2 × 2 × 2 Monkhorst–Pack K-mesh. We chose the CsI-terminated CsPbI_3_ (001) surface because this is the most stable surface with the lowest surface energy, as examined in [34]. A vacuum layer of 18 Å was added in the (001) direction for the surface calculations in order to avoid interactions between the layers. For H_2_O adsorption and insertion into α-CsPbI_3_, the structures were optimized, the internal coordinates fully relaxed, and the lattice parameters fixed. As far as the van der Waals (vdW) forces are concerned, we conducted D3 dispersion correction [35] for the pristine α-CsPbI_3_. The lattice constant was reduced by 0.09 Å, which represents a decrease of around 1.4%. Considering that D3 dispersion correction would increase the binding energy between H and I, which may compensate for the lattice constant reduction, the conclusions drawn in our work are not expected to be affected. Because Cs, Pb, and I are heavy, spin–orbit coupling (SOC) [36] may be significant. As Li et al. [33] have found, SOC mainly decreases the conduction band. Furthermore, it has been found that a GW [37] + SOC calculation can increase the band gap back close to the PBE results. Thus, it seems PBE calculation provides reasonable results for the numerical compensation between GW and SOC.

## 3. Results and Discussion

**Figure 1:**
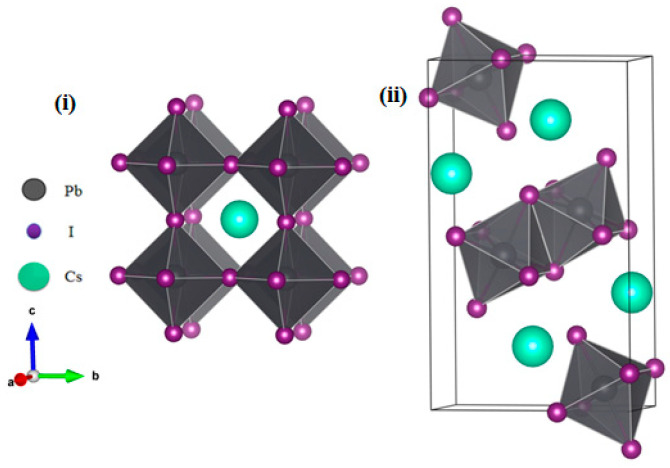
The unit cell structure of α-CsPbI_3_ (**i**) and δ-CsPbI_3_ (**ii**). The Cs, Pb, and I ions are shown as large green, gray, and small purple circles, respectively. The lattice vectors are labeled as a, b, c in (**i**).

The instability of CsPbI_3_ with a highly symmetrical perovskite structure is mainly due to the size mismatch between the constituent ions. In order to stabilize the small Cs in the PbI_6_ octahedral gap, the PbI_6_ octahedron rotates and tilts, resulting in the distortion of the highly symmetrical perovskite structure so as to form less symmetrical non-perovskite structures. Thus, though it possesses better photoelectric properties [19], the cubic structure of CsPbI_3_ is very unstable, and recent experiments and calculations have focused on the instability of α-CsPbI_3_. Therefore, in this work, we focused mainly on the interaction of H_2_O with pristine α-CsPbI_3_ and the intrinsic defects within it. The α- and δ-CsPbI_3_ structures are shown in Figure 1. PbI_6_ extends its three-dimensional framework in an angle-sharing manner along the three coordinate axis directions in α-CsPbI_3_, with a Cs atom in the middle of the eight top corners, a Pb atom in the center of the cubic structure, and an I atom in the six faces of the cube center. PbI_6_ rotates and breaks into δ-CsPbI_3_. As shown, the α-to-δ-CsPbI_3_phase transition involves bond breaking and rebonding [38]. However, the bond rearrangement barrier may be high in the pristine lattice and so the intrinsic defects are expected to play a role in phase transformation [23,26]. H_2_O, α-CsPbI_3_, and the intrinsic defects may also interact.

### 3.1. Comparison of Adsorption of H_2_O on the Surface and in the Bulk of CsPbI_3_

**Figure 2:**
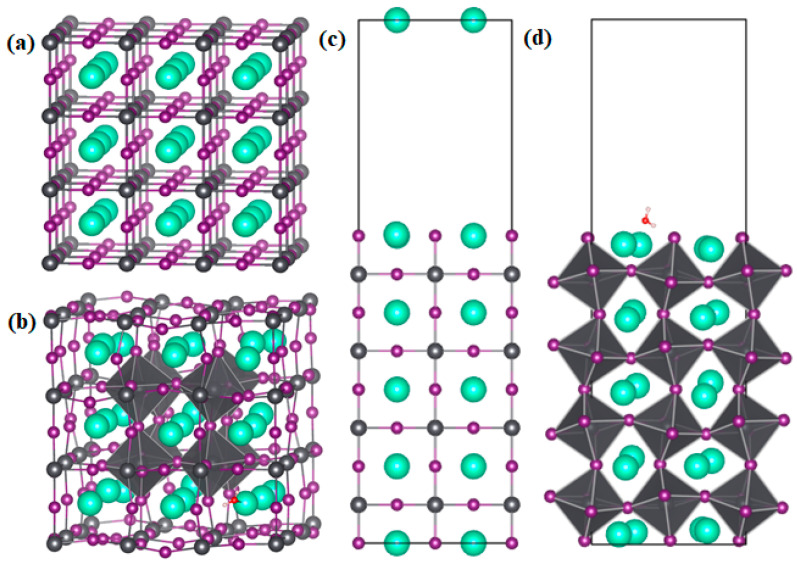
Structures of (**a**,**b**) bulk and (**c**,**d**) (001) surface of α-CsPbI_3_ with and without H_2_O adsorption. The Cs, Pb, and I ions are shown as large green, gray, and small purple circles, respectively. O and H are shown as small red and white circles.

Regarding the position of H_2_O in CsPbI_3_, we first calculated the binding energy *E*_bind_ of H_2_O on the surface and in the bulk of α-CsPbI_3_. Figure 2 shows the structures of the bulk and (001) surface of α-CsPbI_3_ with and without H_2_O adsorption. For H_2_O insertion into the α-CsPbI_3_ bulk, the H_2_O molecule was placed into three different positions, ensuring that O was close to the Cs and that H was close to the I atoms; however, the optimized structure of the three different initial configurations became similar as a result of O binding with Cs and two H atoms binding with I, as can be seen in Figure 2b. The distance between Cs and O is 2.92 Å, and the distance between the two H and I atoms are around 2.65 Å. The binding energy of the three configurations are also similar, as can be seen in Table 1. For H_2_O adsorption on the surface, we first generated the possible configurations that could favor Cs–O and I–H bonds, referencing the configurations in [28]. Similar to this study, the energy difference between the different configurations is small, and we show one of the configurations in Figure 2d. The distance between Cs and O is 3.04 Å, which is a little larger than that in the bulk, and the distance between H and I is around 2.60 Å, which is a little smaller than that in the bulk. The strong coupling between O and Cs and the interaction between H and I led to the strong binding of H_2_O in CsPbI_3_, as in CsSnI_3_ [28]. The binding energy of H_2_O in the bulk and on the surface of α-CsPbI_3_ can be calculated as follows:

(1) $$E_{bind}=E_{ads}-E_{noH_{2}O}-E_{H_{2}O}$$

where $E_{bind}$ represents the binding energy, $E_{ads}$ and $E_{noH_{2}O}$ represent the total energies of the bulk or surface system with and without H_2_O adsorbed, respectively, and $E_{H_{2}O}$ is the total energy of one H_2_O molecule. The energy of the H_2_O molecule was calculated within a cell with a vacuum size of 10 Å to ensure the H_2_O molecule fully separated from its periodic images. The binding energies of H_2_O in the bulk and on the surface of α-CsPbI_3_ are both negative, −1.26 eV and −0.60 eV, respectively, indicating that the binding process is exothermic and can proceed spontaneously, in accordance with prior research on CsSnI_3_. The binding energy is higher in the bulk than that on the surface, even though H_2_O insertion into the bulk may cause a larger distortion, and this could be indicative of the instability of CsPbI_3_, and, thus, CsPbI_3_ could potentially bond with H_2_O. In this regard, H_2_O tends to diffuse into the bulk once it adsorbs on the surface. Thus, we focused on the interaction between H_2_O and the intrinsic defects in the α-CsPbI_3_ bulk in this work.

### 3.2. Effect of Intrinsic Vacancies within the Bulk Phase on H_2_O Insertion

**Figure 3:**
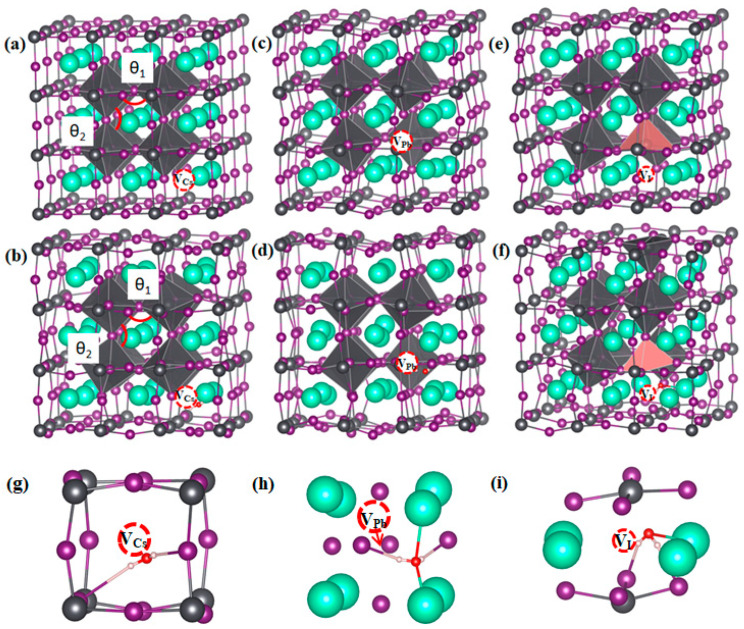
Structures of CsPbI_3_ in the 3 × 3 × 3 supercells (40 atoms) with Cs, I, and Pb vacancies, V_Cs_^−1^, V_I_^+1^, and V_Pb_^−2^ (**a**,**c**,**e**), without and (**b**,**d**,**f**) with H_2_O inserted. The enlarged parts around the vacancies with H_2_O inserted are shown in (**g**–**i**). θ_1_ and θ_2_ represent the 2 I–I–I bond angles between octahedrons close to the vacancies. The atom colors adopted are the same as those in Figure 1. The PbI_6_ octahedron is marked in gray, and the PbI_5_ octahedron is marked in pink. The positions for V_Cs_^−1^, V_I_^+1^, and V_Pb_^−2^ are surrounded by a dashed circle.

**Figure 4:**
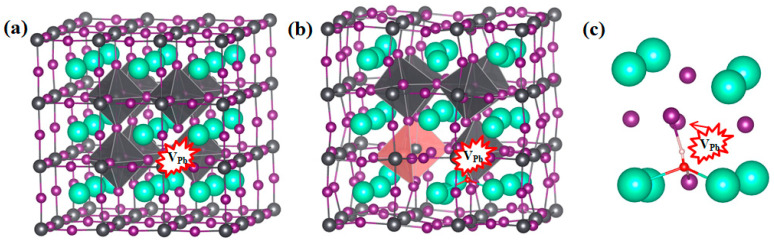
Structures of CsPbI_3_ in the 3 × 3 × 3 supercells (40 atoms) with the Cs, I, and Pb vacancies, V_Pb_^0^, (**a**) without and (**b**) with H_2_O inserted. The enlarged parts around the vacancies with H_2_O inserted are shown in (**c**). The atom and PbI_6_ octahedron colors adopted are the same as those in Figure 3. The positions for V_Pb_^−2^ are surrounded by a red shape.

The distribution of the intrinsic defects can usually be estimated with the concentration formula *c* = *N*_sites_e^−Δ*H*/k*T*^, where *c* represents the concentration of the defect, *N*_sites_ represents the number of sites for the defect per unit volume, Δ*H* represents the formation energy of the defect, and k and *T* represent the Boltzmann constant and temperature, respectively [39]. The formation energies depend on the chemical potentials of the constituent element. As Li et al. [33,40] have calculated, there could be different intrinsic defects within the CsPbI_3_ bulk phase, and they found that the formation energies of Pb, the I vacancy V_Pb_, and V_I_ are extremely low, and even negative, under both Pb/Cs-rich and I-rich conditions. The formation energy of Cs vacancy, V_Cs_, is also relatively low. Thus, the dominant defects in α-CsPbI_3_ are V_Pb_, V_Cs_, and V_I_; moreover, V_Pb_ and V_Cs_ tend to be negatively charged V_Cs_^−1^ and V_Pb_^−2^, respectively, while V_I_ tends to be positively charged V_I_^+1^. Thus, based on a previous study, we studied the interaction of H_2_O with three intrinsic vacancies, V_Cs_, V_Pb_, and V_I_, in α-CsPbI_3_ bulk, and the neutral, V_Cs_^0^, V_Pb_^0^, and V_I_^0^, and charged states, V_Cs_^−1^, V_Pb_^−2^, and V_I_^+1^ [33], were both studied. H_2_O was inserted close to the vacancies, and the relative position for H_2_O with respect to the vacancies can be seen in Figure 3 and Figure 4. The structures given were optimized. The binding energies for H_2_O close to the vacancies can also be calculated with Equation (1), and the results can be seen in Table 2. The long-range Coulomb interactions converge slowly with the supercell size; thus, charge–charge corrections are sometimes needed [39]. We addressed the energy difference before and after H_2_O insertion and, thus, the electrostatic energy from the spuriously repeated charges was expected to be canceled out. Moreover, this process has not been conducted when calculating the 3 × 3 × 3 supercells of α-CsPbI_3_ [33]. Therefore, charge–charge correction was neglected in this work. As shown, and compared with that in the pristine lattice, the binding energy of H_2_O near the charged vacancies, V_Cs_^−1^, V_I_^+1^, and V_Pb_^−2^, decreased or was almost unchanged. The binding energy of H_2_O near the neutral vacancies, V_I_^0^ and V_Cs_^0^, further decreased, which could be attributed to the decreased charge on the atoms near V_I_^0^ and V_Cs_^0^, which in turn reduced the Coulomb attraction between I/Cs and H_2_O, which were responsible for the binding between H_2_O and CsPbI_3_ [28]. However, the introduction of V_Pb_^0^ significantly increased the binding energy of H_2_O. We then analyzed the changes in the structures in these systems. It must be noted that no matter which vacancy was introduced, the insertion energy was always negative, indicating that the insertion of H_2_O is always an exothermic process and can spontaneously occur. 

**Figure 5:**
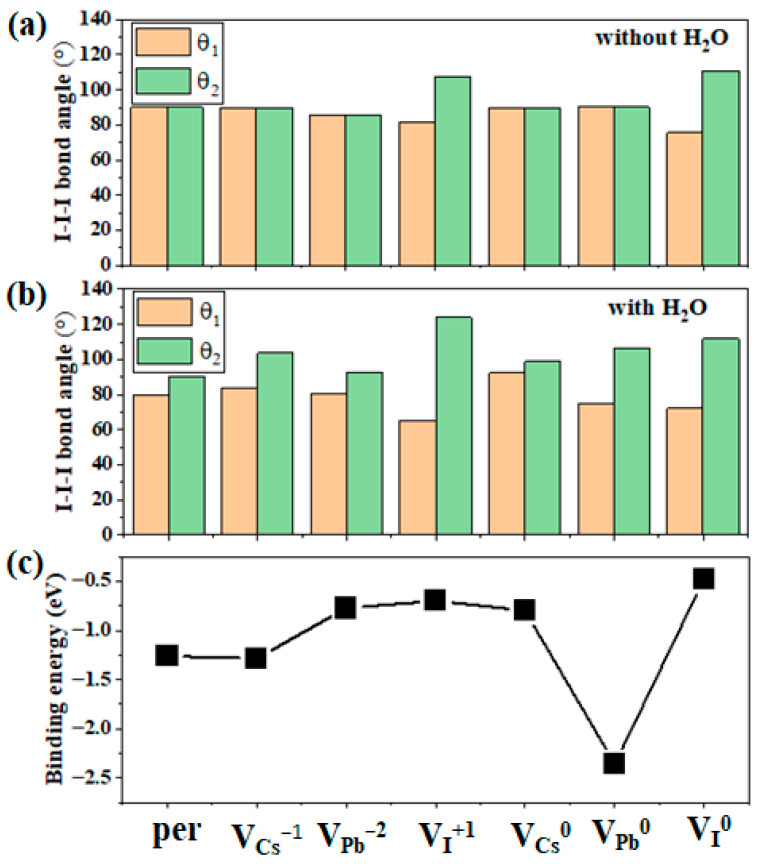
I–I–I bond angles θ_1_ and θ_2_ between octahedrons close to the position of H_2_O, as marked in Figure 3, (**a**) before and (**b**) after H_2_O insertion. (**c**) Binding energies for H_2_O in CsPbI_3_, including the vacancies and the pristine lattice.

Figure 3 shows the optimized structure of α-CsPbI_3_ with V_Cs_^−1^, V_I_^+1^, and V_Pb_^−2^ with or without H_2_O inserted. As shown, when only V_Cs_^−1^ or V_Pb_^−2^ was introduced, the original cubic structure was not significantly distorted, and the octahedron did not significantly rotate or twist. In the vicinity of the V_Cs_^−1^ vacancy, due to the removal of the attraction of Cs, I slightly moved away from the vacancy, the nearest Pb–I bond was slightly bent, and the remaining I atoms and all of the Pb and Cs atoms are located in their original highly symmetrical position without being offset. For V_Pb_^−2^, compared with the perfect supercell, the bond length of I–Pb adjacent to V_Pb_^−2^ was reduced by about 0.1 Å; however, the I–Pb–I bond angle was basically unchanged. Thus, the octahedron framework did not deviate from the perfect supercell, and only adjacent Cs was slightly shifted towards V_Pb_^−2^. However, when V_I_^+1^ was introduced, the PbI_6_ octahedron rotated and twisted to a large extent, and Cs also deviated from the center and moved away from V_I_^+1,^ due to the removal of the attraction or repulsion of the I. The large distortion induced by V_I_^+1^ is consistent with the results of Lin et al. [23], who found that V_I_^+1^ in the crystal lattice can effectively catalyze the transformation from the α to δ phases. However, when H_2_O is introduced to V_Cs_^−1^ and V_Pb_^−2^, the structures in the vicinity of the vacancies become significantly distorted. Moreover, H_2_O adsorption onto V_I_^+1^ contributes to further distortion in the vicinity of V_I_^+1^, thus enhancing its effect on phase transformation. To manifest the rotation or twisting of the octahedron, we measured the 2 I–I–I bond angles between the octahedrons close to the vacancies, θ_1_ and θ_2_, as labeled in Figure 3, and the results are shown in Figure 5. As shown, θ_1_ and θ_2_ were almost 90° when only V_Cs_^−1^ or V_Pb_^−2^ was introduced, and they shifted away from 90° when only V_I_^+1^ was introduced. Additionally, θ_1_ and θ_2_ shifted away from 90° when H_2_O was then introduced to V_Cs_^−1^ or V_Pb_^−2^, even though this shift is still smaller than that for only V_I_^+1^. Another shift occurred on θ_1_ and θ_2_ when H_2_O was further introduced to V_I_^+1^, indicating that H_2_O adsorption onto V_I_^+1^ further contributes to the effect of V_I_^+1^ on the phase transition of CsPbI_3_.

**Figure 6:**
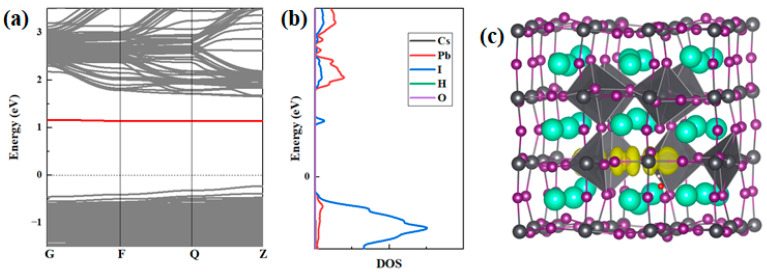
(**a**) The band structure and (**b**) the projected density of state (PDOS) for CsPbI_3_ in the 3 × 3 × 3 supercells (40 atoms) with neutral Pb vacancies V_Pb_^0^, with H_2_O inserted. The defect level in (**a**) in the band gap is shown in red. The PDOSs for Cs, Pb, I, H, and O are given in different colors in (**b**). (**c**) The partial charge densities of the defect levels are shown in red (**a**). The atom colors adopted are the same as in Figure 1. The isosurface of the partial charge density is shown in yellow.

On the other hand, for the neutral state, the distortion induced by H_2_O adsorption onto V_Cs_^0^ and V_I_^0^ is smaller compared with their charged states, while the distortion induced by H_2_O adsorption onto V_Pb_^0^ is larger, which is in accordance with the insertion energy trend. Thus, we focused on the effect of H_2_O adsorption onto V_Pb_^0^. Figure 4 shows the optimized structures of V_Pb_^0^ before and after inserting H_2_O near V_Pb_^0^. As shown, when we introduced only V_Pb_^0^ to α-CsPbI_3_, the symmetrical structure of α-CsPbI_3_ remained basically unchanged. However, when we inserted H_2_O near V_Pb_^0^, a huge distortion of the structure was induced, and this distortion is even comparable with the distortion induced by V_I_^−^. An important feature is that some of the PbI_6_ octahedrons actually “disintegrated”, with the measured distances between Pb and I increasing from 3.21 Å to 3.9 Å and 3.1 Å to 4.06 Å, respectively, and the two I atoms actually formed new I–I bonds. The Pb–I bond broke, and a new stable I–I bond formation may be the dominant reason for the significantly high binding energy for H_2_O inserted near V_Pb_^0^. To absolutely characterize the bonding nature for H_2_O near V_Pb_^0^, we conducted band structure and charge density calculations, as can be seen in Figure 6. We found that, as shown in Figure 6a, an isolated energy level formed, and that the corresponding charge was indeed located between the I atoms, as seen in Figure 6b,c. The disintegrated PbI_6_ octahedrons and new I–I bond formation indicate that the effect of H_2_O on V_Pb_^0^ could equivalently be recognized as formation of two I vacancies, V_I_, close to the one Pb vacancy, V_Pb_. The large distortion induced is consistent with the effect of the V_I_ vacancies and, thus, may also promote α-to-δ-phase transformation. In addition, the binding energy of H_2_O near V_Pb_^0^ is around 2 eV higher than that of V_Pb_^2−^, and the formation energy of V_Pb_^2−^ is only nearly 2 eV lower than that of V_Pb_^0^ near the Fermi level, as can be found in [33]; thus, V_Pb_^2−^ probably causes the transformation from V_Pb_^2−^ to V_Pb_^0^ and binds strongly with H_2_O if H_2_O is inserted. Considering its abundancy, the transformation from V_Pb_^2−^ to V_Pb_^0^ and the strong binding with H_2_O are expected to cause H_2_O to catalyze the α-to-δ phase transformation of CsPbI_3_.

**Figure 7:**
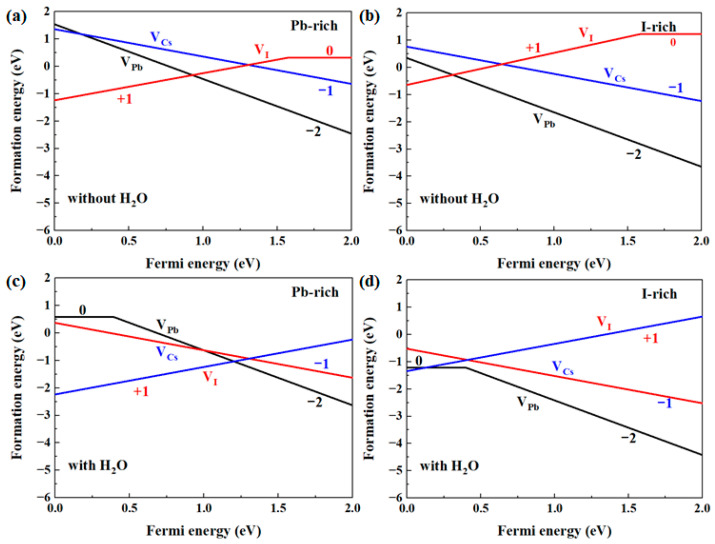
Formation energies of V_Pb_, V_Cs_, and V_I_ in α-CsPbI_3_ at high- and low-Pb-level conditions (**a**,**b**) without and (**c**,**d**) with H_2_O. The charges on the defects are labeled alongside each segment.

To further illustrate the interaction between the defects and H_2_O, we calculated the formation energies of V_Cs_, V_Pb_, and V_I_ in α-CsPbI_3_ with and without H_2_O insertion, as shown in Figure 7. The formation energy is calculated with the following equation [39,41,42,43,44,45]:

(2) $$∆H_{f}\left(q\right)=E\left(q\right)-E\left(pristine\right)+\sum_{i}n_{i}\left[\mu_{i}+E\left(i\right)\right]+q\left[E_{F}+\varepsilon_{VBM}\right]$$

where *E*(*q*) represents the energy of the defect system in charged state *q*, *E*(pristine) represents the energy of the pristine system, *ε*_VBM_ represents the energy of the valence band maximum (VBM), and *E*_F_ represents the Fermi energy in reference to *ε*_VBM_. *n_i_* represents the number of atoms *i* added (*n_i_* < 0) or removed (*n_i_* > 0) from the system, *E*(*i*) represents the energy of the element solid *i*, and *μ_i_* represents the chemical potential in reference to *E*(*i*). The choice of *E*(*i*) for Cs, Pb, and I were the same as those in [33,40], and the *E*(*i*) for H_2_O represents the energy of one H_2_O molecule. 

As shown, the formation energies of the defects without H_2_O insertion are generally consistent with those in [33]. We compared the formation energies with and without H_2_O insertion. As shown, H_2_O insertion reduces the formation energy of the defects due to binding between H_2_O and the different defects. Specifically, the formation energy of V_Pb_^0^ could become lower than that for V_Pb_^2−^ when the Fermi energy is near the VBM, and this is in accordance with the estimation we derived from binding energy analysis, in which V_Pb_^2−^ is able to transform into V_Pb_^0^. Regarding the large deformation promoted in V_Pb_^0^, the formation of V_Pb_^0^ is expected to be capable of promoting phase transition. Moreover, the formation of V_Pb_^0^ tends to occur close to the VBM; the p-type samples synthesized under low-Pb-level conditions are expected to be affected more. We estimated the effect of charge–charge correction [42,44,45]. The charge–charge interaction between the periodic images could be estimated in the form ~*q*^2^/4π*εL* [46], where *q* represents the charge on the defect, *L* represents the lateral size of the supercell, and *ε* represents the relative static dielectric constant, which is around six in α-CsPbI_3_ [47]. We took V_Pb_^2−^ with the higher valence of −2 for the estimation, and the interaction energy is around 0.2 eV within the 3 × 3 × 3 supercell. This correction may move the 0/−2 transition level of V_Pb_ by around 0.1 eV; however, the main conclusions state that V_Pb_^2−^ can transform into V_Pb_^0^ when the Fermi energy located near the VBM does not change. On the other hand, we mainly compared the formation energy of the defects before and after H_2_O insertion in this work; the electrostatic energies from the spuriously repeated charges were expected to be canceled out. Moreover, regarding the defect levels of V_Pb_ that reside within the band gap between the unoccupied conduction bands, the main conclusion in this work is expected to be unaffected by the band filling effect [48]. 

**Figure 8:**
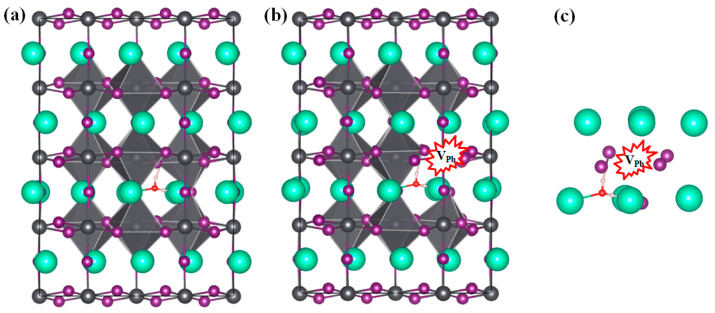
Structures of γ-CsPbI_3_ (**a**) without and (**b**) with Pb vacancy V_Pb_^0^, and with H_2_O inserted. The enlarged parts around the vacancy with H_2_O inserted are shown in (**c**). The atom and PbI_6_ octahedron colors adopted are the same as those in Figure 3. The positions for V_Pb_^−2^ are surrounded by a red shape.

It must be noted that polymorphous symmetry breaking may occur in α-CsPbI_3_, where α-CsPbI_3_ possesses a polymorphous network arranged with local structural motifs of the low-temperature phase with low-level symmetry [24,49]. However, the dominant defects in the low-temperature β- or γ-CsPbI_3_ are also V_Pb_, V_I_, and V_Cs_, as calculated in the previous literature [32]; thus, the defects that we chose to study in this work are reasonable. We conducted further calculations to explore the effect on the defect formation energy. In this work, we found that the dominant role that the defects may play in phase transition when H_2_O is present is that H_2_O can promote both the rearrangement of I atoms into PbI_6_ octahedrons and the Pb–I bond breakage nearest to V_Pb_^0^, which can eventually promote phase transition. We then conducted a calculation for H_2_O insertion into V_Pb_^0^ in γ-CsPbI_3_. However, as can be seen in Figure 8, no significant bond breakage or I atom rearrangements were found. This indicates that whether V_Pb_^0^ takes effect indeed depends on the surrounding structures, and that limited deformation can occur in the low-level symmetry system, which is consistent with its higher stability. However, the cubic structure can possess more local deformations [24], revealing its higher flexibility. As a result, this depends on whether V_Pb_^0^ in the cubic phase with local deformation can be deformed by H_2_O insertion. Moreover, there may be fluctuation in the local environment of V_Pb_^0^ in the cubic phase, which may deserve further study.

## 4. Conclusions

In this paper, first-principles calculations based on the density functional theory are used to theoretically study the interaction between H_2_O and CsPbI_3_, including the pristine lattice and intrinsic vacancies therein, and the α-to-δ phase transition of CsPbI_3_ catalyzed by H_2_O is analyzed. First, we compared the binding of H_2_O on the surface and in the bulk of α-CsPbI_3_, and the binding energy is higher in the bulk than it is on the surface, at −1.26 and −0.60 eV, respectively. Thus, H_2_O tends to diffuse into the bulk once it adsorbs on the surface. We further studied the interaction between three intrinsic vacancies, V_Pb_, V_I_, and V_Cs_, in the α-CsPbI3 bulk and H_2_O insertion. It was found that the insertion energy of H_2_O decreased or was almost unchanged upon the insertion of the charged vacancies, V_Cs_^−1^, V_I_^+1^, and V_Pb_^−2^, and neutral vacancies, V_I_^0^ and V_Cs_^0^, while the introduction of V_Pb_^0^ significantly increased the binding energy of H_2_O and, thus, promoted the insertion of H_2_O into the lattice. The strong binding between H_2_O and V_Pb_^0^ induced Pb–I bond breakage and new I–I bond formation, which are expected to play roles in the phase transition from α-CsPbI_3_ to δ-CsPbI_3_. Moreover, H_2_O adsorption onto V_I_^+1^ contributes to a larger distortion in the vicinity of V_I_^+1^, which is expected to enhance the phase transition effect of V_I_^+1^. The result is expected to provide guidance for the improvement of the stability of α-CsPbI_3_, especially in humid environments.

## Figures and Tables

**Table 1:** Binding energy of H_2_O in the bulk of α-CsPbI_3_ at different initial positions.

Initial Position of H_2_O Insertion	Binding Energy *E*_bind_/eV
near Cs	−1.21
near Pb	−1.25
near I	−1.26

**Table 2:** Binding energy of H_2_O near different vacancies in the bulk of α-CsPbI_3_.

Position of H_2_O Insertion	Binding Energy *E*_bind_/eV
Near V_Cs_^−1^	−1.29
Near V_Pb_^−2^	−0.78
Near V_I_^+1^	−0.70
Near V_Cs_^0^	−0.80
Near V_Pb_^0^	−2.36
Near V_I_^0^	−0.48

## Data Availability

The original contributions presented in the study are included in the article, further inquiries can be directed to the corresponding author.
